# Differentiated effect of hypothermia versus normothermia after cardiac arrest by neurological status and clinical correlates of ischemic burden

**DOI:** 10.1186/s13054-026-06414-x

**Published:** 2026-11-02

**Authors:** Gabriele Lileikyte, Lucas L. Marinho, Ying Lu, Philippe Boileau, Marion Moseby-Knappe, Alexandra Spicer, Josef Dankiewicz, Gisela Lilja, Matt P. Wise, Claudia Schrag, Anders Aneman, Matthias Haenggi, Anders M. Grejs, Janneke Horn, Pascal Stammet, Janus C. Jakobsen, Jesper Kjaergaard, Christian Rylander, Michael O. Harhay, Matthew M. Churpek, Niklas Nielsen, Patrick R. Lawler

**Affiliations:** 1 Anesthesiology and Intensive Care, Department of Clinical Sciences Helsingborg, Lund University, Svartbrödragränden 3, Helsingborg, 251 87 Sweden; 2 Department of Intensive and Perioperative Care, Skåne University Hospital, Malmö, Sweden; 3 Cardiology and Critical Care Medicine, McGill University Health Centre, McGill University, Montréal, Canada; 4 Departments of Epidemiology, Biostatistics and Occupational Health, McGill University, Montréal, Canada; 5 Neurology and Rehabilitation Medicine, Department of Clinical Sciences Lund, Lund University, Lund, Sweden; 6 Department of Rehabilitation Medicine, Skåne University Hospital, Lund, Sweden; 7 Department of Medicine, University of Wisconsin-Madison School of Medicine and Public Health, Madison, Wisconsin, WI USA; 8 Cardiology, Department of Clinical Sciences Lund, Lund University, Lund, Sweden; 9 Department of Cardiology, Skåne University Hospital, Lund, Sweden; 10 Neurology, Department of Clinical Sciences Lund, Lund University, Lund, Sweden; 11 Department of Neurology, Skåne University Hospital, Lund, Sweden; 12 Adult Critical Care Unit, University Hospital of Wales, Cardiff, UK; 13 Division of Intensive Care Medicine, HOCH Cantonal Hospital St. Gallen, St. Gallen, Switzerland; 14 Intensive Care Unit, Liverpool Hospital, Sydney, Australia; 15 South Western Sydney Clinical School, University of New South Wales, Sydney, Australia; 16 Institute of Intensive Care Medicine, University Hospital Zurich, University of Zurich, Zurich, Switzerland; 17 Department of Intensive Care Medicine, Aarhus University Hospital, Aarhus, Denmark; 18 Department of Clinical Medicine, Aarhus University, Aarhus, Denmark; 19 Department of Intensive Care, Amsterdam Neurosciences, Amsterdam University Medical Center, Amsterdam, The Netherlands; 20 Department of Anesthesia and Intensive Care Medicine, Centre Hospitalier de Luxembourg, Luxembourg City, Luxembourg; 21 Department of Health, Medicine and Life Sciences, Faculty of Science, Technology and Medicine, University of Luxembourg, Esch-sur-Alzette, Luxembourg; 22 Department of Regional Health Research, Faculty of Health Sciences, University of Southern Denmark, Odense, Denmark; 23 Copenhagen Trial Unit, Centre for Clinical Intervention Research, Copenhagen University Hospital, Copenhagen, Denmark; 24 Department of Cardiology, The Heart Center, Copenhagen University Hospital, Copenhagen, Denmark; 25 Department of Clinical Medicine, Faculty of Health Science, University of Copenhagen, Copenhagen, Denmark; 26 Anesthesia and Intensive Care, Department of Surgical Sciences, Uppsala University, Uppsala, Sweden; 27 Department of Biostatistics, Epidemiology and Informatics, Department of Medicine, Perelman School of Medicine, University of Pennsylvania, Philadelphia, PA USA; 28 Anesthesiology and Intensive Care, Department of Clinical Sciences Lund, Lund University, Lund, Sweden; 29 Department of Intensive and Perioperative Care, Skåne University Hospital, Lund, Sweden; 30 Cardiology and Critical Care Medicine, University of Toronto, Toronto, Canada

**Keywords:** Heterogeneity of treatment effect, Targeted temperature management, Therapeutic hypothermia, Out-of-hospital cardiac arrest, Heart arrest

## Abstract

**Introduction:**

The optimal temperature target after out-of-hospital cardiac arrest (OHCA) remains uncertain, as major randomized trials have not demonstrated a clear benefit of lower-target hypothermia compared with normothermia. Despite this, patients with differing degrees of ischemic burden or early neurological injury may hypothetically respond differently to temperature targets, raising the possibility of heterogeneous treatment effects. This study aimed to determine whether outcomes differ after OHCA with 33 °C versus controlled normothermia (36.0–37.7 °C) according to correlates of ischemic burden and neurological findings at hospital admission.

**Methods:**

This secondary analysis of the TTM and TTM2 trials employed effect-based machine learning to probe for heterogeneous treatment effects. Causal forests and Bayesian additive regression trees (BART) estimated individualized treatment effects (ITE) based on ischemia duration, prehospital factors, and neurological examination findings at hospital admission. Treatment effect modifier variable importance parameter (TEM-VIP) analysis ranked the baseline variables most likely to modify effect. The primary outcome was six-month all-cause mortality, and the secondary outcome was poor functional outcome (modified Rankin scale score 4–6). Primary analyses evaluated potential heterogeneity within each trial, while secondary analyses pooled all participants.

**Measurements and main results:**

All 2789 adult OHCA patients from the trials’ primary analyses were included (median age 65 years (IQR 56–73), 79.9% male). Death occurred in 49.4% of patients in the 33 °C group, and in 47.9% in the controlled normothermia group. Variation in six-month mortality ITEs estimated from ischemic burden correlates and initial neurological findings was minimal, and no significant heterogeneity of treatment effect was identified across all methods and analysis populations. In the pooled analysis, estimated conditional mean mortality risk difference ranged from − 1.2% to 2.1% in causal forest (*p =* 0.99), and from − 0.5% to 1.5% in BART, with posterior probabilities for hypothermia benefit ranging from 42% to 53%. Absolute subgroup estimates in TEM-VIP analysis were small and non-significant. Results were consistent for the six-month functional outcome.

**Conclusions:**

Among TTM and TTM2 participants randomized to 33 °C versus controlled normothermia post-OHCA, no evidence of detectable heterogeneity of treatment effect based on available clinical correlates of ischemic burden or initial neurological findings was identified, with minimal estimated variation in treatment effect.

**Supplementary Information:**

The online version contains supplementary material available at https://doi.org/10.1186/s13054-026-06414-x.

## Introduction

Whether temperature control to low-target hypothermia influences survival and neurological function in select patients following out-of-hospital cardiac arrest (OHCA) remain uncertain. Clinical practice guidelines reflect this uncertainty: the American Heart Association guidelines recommend target temperatures within a broad range of 32 °C to 37.5 °C, whereas the European Resuscitation Council advises actively preventing fever by keeping the temperature below 37.5 °C [1, 2]. Two large randomized clinical trials (RCTs) – the Target Temperature Management at 33 °C versus 36 °C after Cardiac Arrest (TTM) and the Hypothermia versus Normothermia after Out-of-hospital Cardiac Arrest (TTM2) – observed no significant differences in outcomes between targeted hypothermia (33 °C) and controlled normothermia strategies [3–5]. However, it has been hypothesized that patients with differing degrees of ischemic burden and early neurological injury may respond differently to temperature targets [6–9]. Specifically, patients with clinical signs of more severe neurological injury on admission have been proposed as a subgroup that may derive greater benefit from 33 °C, hypothesizing heterogeneous treatment effects (HTE) [10]. Consistent with this hypothesis, observational data have suggested lower temperature targets may be associated with improved neurological outcomes in patients with moderate disease severity, a hypothesis currently being evaluated in an ongoing RCT [11, 12]. Prior single-variable subgroup analyses in TTM and TTM2 failed to identify consistent evidence of HTE [3–5]. These analyses, however, have important methodological limitations. Continuous variables typically require categorization using arbitrary thresholds, patients may belong to multiple overlapping subgroups, and treatment effects are generally evaluated one characteristic at a time. Consequently, conventional subgroup analyses may fail to detect heterogeneity arising from complex interactions among multiple patient characteristics and may have limited statistical power to identify clinically relevant effect modification [13].

Recently, effect-based modeling, which estimates how treatment effects vary across individuals rather than assuming a single average effect, has been increasingly deployed to assess potential HTE in critical care trials [14, 15]. By evaluating treatment effects across multiple patient characteristics simultaneously without requiring prespecified subgroup definitions, these approaches may detect patterns of effect modification that are difficult to identify using conventional subgroup analyses. Given ongoing clinical uncertainty whether patients with clinical signs of greater ischemic burden or worse initial neurological findings benefit differently from lower-target temperature control, we conducted a *post hoc* secondary analysis of the TTM and TTM2 trials to explore potential HTE in temperature targets after OHCA. Accordingly, we aimed to evaluate if clinical variables that correlate to more extensive ischemic burden or initial signs of more profound neurological injury exhibited differential responses to a 33 °C temperature target using effect-based machine learning methods.

## Methods

### Patient populations and data sources

The TTM (NCT01020916) and TTM2 (NCT02908308) trial designs and primary findings have been previously published [3, 4, 16, 17]. The present secondary analysis included all patients from the primary analysis populations of the trials (939/950 randomized patients in TTM and 1850/1900 in TTM2, Supplementary Figure S1). Briefly, these international, parallel-group, open-label RCTs enrolled adults resuscitated from OHCA due to a presumed cardiac cause in TTM and a presumed cardiac or unknown cause in TTM2. Full eligibility criteria are presented in Supplementary Table S1. Both trials randomized patients to either targeted hypothermia at 33 °C for 28 h, or to normothermia. Normothermia was defined as a target temperature of 36 °C in the TTM trial and as maintenance of normothermia with treatment of fever (≥ 37.8 °C) in the TTM2 trial. Recruitment was allowed within 240 min of return of spontaneous circulation (ROSC) in TTM and within 180 min in TTM2. The trials were approved by the relevant ethics committees in each participating country. Informed consent was initially waived, deferred, or obtained from a legal surrogate, and later obtained from patients who regained capacity. No separate ethics approval was required for the pooled secondary analyses, which were conducted using de-identified trial data in alignment with the original trial approvals.

### Clinical covariates

To evaluate whether correlates of ischemic burden and baseline neurological findings modify the relative treatment effect of therapeutic temperature targets, variables reflecting pre-hospital arrest, resuscitation, and initial neurological status were selected. In total, 11 available trial-collected variables were included in the analysis. Variables reflecting ischemic burden included time to initiation of advanced life support (ALS), time to ROSC, bystander cardiopulmonary resuscitation (CPR), initial shockable cardiac rhythm, and location of cardiac arrest. These variables were selected as clinically available correlates of ischemia-reperfusion injury rather than direct measures of the underlying biological injury process. Baseline neurological findings that were recorded upon hospital admission included motor and eye responses from the Full Outline of UnResponsiveness (FOUR) score, spontaneous breathing (absent/present), pupillary and corneal reflexes (bilaterally absent or present), and the presence of generalized myoclonus [18]. Selection was guided by the clinical hypothesis that targeted temperature management may provide the greatest benefit to patients with clinical signs of both a more extensive ischemic burden and severe early neurological impairment [10, 11, 19, 20]. Accordingly, the analysis focused on a prespecified set of admission variables considered most relevant to this hypothesis and available at the time of temperature target selection. Assessment methods for baseline neurological findings differed between TTM and TTM2 trials; details on the harmonization of variables between trials are provided in Supplementary Methods. Missing data were imputed using a non-parametric Random Forest-based algorithm (*missForest*) [21], which accommodates both continuous and categorical variables without distributional assumptions; full details of the imputation procedure are provided in the Supplementary Methods.

### Clinical outcomes

Consistent with the outcomes used in the original TTM and TTM2 trials, the primary outcome was all-cause mortality at six months, and the secondary outcome was poor functional outcome at six months, defined as modified Rankin scale score of 4 to 6 (moderately severe disability, severe disability, or death) [22]. Both outcomes were analyzed as landmark endpoints.

### Effect-based individualized treatment effect estimation

Effect-based machine learning involves building prediction models that estimate each patient’s predicted outcome with and without the investigational treatment. These models are based on the provided baseline variables and on randomized group allocation. The difference between the predicted outcomes with treatment and control yields an individual-level estimate of the conditional average treatment effect (CATE). These patient-specific treatment effects were referred to as individualized treatment effect (ITE) estimates throughout the manuscript. We used two approaches to estimate individual-level CATEs for each trial participant: causal forests and Bayesian additive regression trees (BART) causal machine learning models. The resulting ITE estimates were used in subsequent analyses of heterogeneity of treatment response.

First, we applied causal forests, a derivative of the random forests algorithm [23], which builds prediction trees that iteratively split observations into covariate-based subgroups with maximally different average treatment effect estimates in the predicted outcomes between the treatment and control [24, 25]. We chose causal forests because they provide a flexible, nonparametric approach that offers asymptotically consistent estimates of ITE. Causal forests were fitted using Generalized Random Forest (*gfr*) package (version 2.4.0) in R [25]. Estimates were obtained using sample-splitting and orthogonalization to reduce bias, and the presence of HTE was formally evaluated using an omnibus calibration test [26]. To evaluate the robustness of the causal forest-derived ITEs, we additionally performed nested outer cross-validation (Supplementary Methods).

Second, we applied BART, a non-parametric Bayesian tree-based ensemble that estimates ITEs by modeling posterior contrasts between treatment and control [27]. BART provides robust, regularized estimates of treatment effects and quantifies uncertainty through credible intervals, performing reliably even with complex nonlinearities and interactions. BART was implemented using a custom parallelized framework based on the *lbart* function from the *BART* R package (version 2.9.9) [28]. The presence of HTE was assessed by examining the variation of posterior probabilities of beneficial treatment effect. Additional details on model specification and tuning for causal forests and BART are provided in the Supplementary Methods.

To ensure that no significant effect modifiers had been overlooked, we performed a treatment effect modifier variable importance parameter (TEM-VIP) analysis to identify and rank baseline variables most likely to modify treatment effect [29, 30]. The TEM-VIP of a given baseline variable reports the expected change in the average treatment effect for a unit increase in said variable. TEM-VIP inference relies on causal machine learning estimators, like targeted maximum likelihood estimators [31, 32], which can incorporate an ensemble of flexible machine learning models [33] to quantify and test each variable’s contribution to potential HTE. This method served as a complementary variable-ranking approach to assess whether any baseline covariates demonstrated evidence of treatment-effect modification. Full details are available in the Supplementary Methods.

### Secondary analyses

The primary analytic approach fitted models independently in TTM and TTM2 trial datasets. As a secondary exploratory analysis, models were additionally fitted in a single cohort comprising all TTM and TTM2 patients. This analysis examined whether signals of HTE could be identified in a larger patient-level dataset while accounting for trial membership as a covariate. Given differences in comparator strategies, eligibility criteria, and trial conduct between TTM and TTM2 (Supplementary Table S1), pooled analyses were considered supportive rather than confirmatory.

Prior single-variable subgroup analyses evaluating several of the included variables did not identify consistent effect modifiers in TTM and TTM2 [5]. To further ensure that no significant effect modifiers were missed, we performed single-variable subgroup analysis of correlates of ischemic burden and neurological variables not previously evaluated in prior studies (cardiac arrest location, pupillary reflexes, corneal reflexes, spontaneous breathing, FOUR motor score, FOUR eye score, and generalized myoclonus). Analyses were conducted using Poisson regression with a log link function and robust standard errors [34]. Additional method details are available in the supplementary methods.

To assess robustness to missing data, complete case sensitivity analyses were conducted in the pooled cohort by restricting analyses to participants with complete data for all model variables (Supplementary Methods).

All statistical analyses were performed using R statistical computing software version 4.5.1 [35]. The figures were prepared using GraphPad Prism version 10.5.0 for Mac, GraphPad Software, Boston, Massachusetts USA. For analyses involving p-values, statistical significance was defined as one-sided α = 0.05.

## Results

The TTM trial enrolled between 2010 and 2013 (*n* = 950) and TTM2 trial between 2017 and 2020 (*n* = 1900). The present analysis included all patients from the primary analysis populations of the parent trials (939 patients from TTM and 1850 patients from TTM2), corresponding to 2789 participants overall (Supplementary Figure S1). Median age was 65 (IQR 56–73) years, and 79.9% were male. Overall, 1398 patients were assigned to 33 °C and 1391 patients to controlled normothermia. Patient characteristics, including variables reflecting ischemic burden and baseline neurological findings, were similar between the intervention groups (Supplementary Table S4); there were modest differences in several prehospital arrest characteristics and baseline neurological traits between the two RCTs (Table 1). Death by 6-months occurred in 691/1398 (49.4%) patients assigned to 33 °C, and in 666/1391 (47.9%) assigned to normothermia.

**Table 1:** Patient characteristics

	TTM (*N* = 939)	TTM2 (*N* = 1850)
Numbers of patients in the present analysis vs. randomized to original trial	939/950 (98.8%)	1850/1900 (97.3%)
Age in years	65.0 (56.5–73.0)	65.0 (56.0–74.0)
Male sex	761 (81.0%)	1467 (79.3%)
**Medical history (known/previous)**		
Heart failure	61/936 (6.5%)	182/1761 (10.3%)
Acute myocardial infarction	193/937 (20.6%)	291/1761 (16.5%)
Coronary-artery bypass grafting	89/935 (9.5%)	149/1761 (8.5%)
Hypertension	374/935 (40.0%)	642/1761 (36.5%)
**Cardiac arrest**		
Cardiac arrest at home	500/938 (53.3%)	977/1850 (52.8%)
Minutes to ALS	9.0 (6.0–13.0) †	10.0 (6.0–15.0)
Bystander CPR performed	683/937 (72.9%)	1477 (79.8%)
Initial rhythm shockable	752/938 (80.2%)	1360 (73.5%)
Minutes to ROSC	25.0 (17.0–39.0) ‡	25.0 (17.0–40.0)
**Clinical characteristics on admission**		
Pupillary reflexes present	704/915 (76.9%)	1072/1528 (70.2%)
Corneal reflexes present	520/796 (65.3%)	373/1043 (35.8%)
Spontaneous breathing present	599/901 (66.5%)	452/1722 (26.2%)
Motor response, FOUR §		
No response to pain (0)	491 (52.3%)	1452 (78.5%)
Extension response to pain (1)	39 (4.2%)	54 (2.9%)
Flexion response to pain (2)	107 (11.4%)	113 (6.1%)
Localizing to pain (3)	23 (2.4%)	61 (3.3%)
Thumbs-up, fist, or peace sign (4)	1 (0.1%)	5 (0.3%)
Eye response, FOUR **		
Eyelids remain closed with pain (0)	624 (66.5%)	1560 (84.3%)
Eyelids closed but open to pain (1)	15 (1.6%)	43 (2.3%)
Eyelids closed but open to loud voice (2)	3 (0.3%)	6 (0.3%)
Eyelids open but not tracking (3)	0 (0%) ***	53 (2.9%)
Eyelids open and tracking (4)	10 (1.1%)	8 (0.4%)
Generalized myoclonus present	35/936 (3.7%)	43/1578 (2.7%)
Initial temperature (°C) ††	35.4 (34.6–36.0)	35.5 (34.8–36.1)
pH ‡‡	7.23 (7.11–7.31)	7.21 (7.10–7.29)
Lactate mmol/l §§	6.0 (3.0-9.5)	4.8 (2.6–8.1)
Circulatory shock	137/937 (14.6%)	534/1850 (28.9%)
ST-segment elevation myocardial infarction	384/928 (41.4%)	742/1828 (40.6%)

* Results reported as numbers (percentages) or median (interquartile range), as appropriate. TTM denotes Targeted Temperature Management at 33 °C versus 36 °C after Cardiac Arrest trial; TTM2, Hypothermia versus Normothermia after Out-of-Hospital Cardiac Arrest trial; ALS, advanced life support; CPR, cardiopulmonary resuscitation; ROSC, return of spontaneous circulation; FOUR, Full Outline of Unresponsiveness Score

† Missing values in 17 patients

‡ Missing value in one patient

§ Missing values in 278 patients for the TTM trial and 165 patients for the TTM2 trial

** Missing values in 287 patients for the TTM trial and 180 patients for the TTM2 trial

*** FOUR score for TTM trial was created from available Glasgow Coma Scale score values that do not include FOUR 3 (Eyelids open but not tracking)

†† Missing values in 36 patients for the TTM trial and 300 patients for the TTM2 trial

‡‡ Missing values in 45 patients for the TTM trial and 33 patients for the TTM2 trial

§§ Missing values in 62 patients for the TTM trial and 80 patients for the TTM2 trial

### Individualized treatment effects derived from variables reflecting ischemic burden and neurological traits

**Fig. 1:**
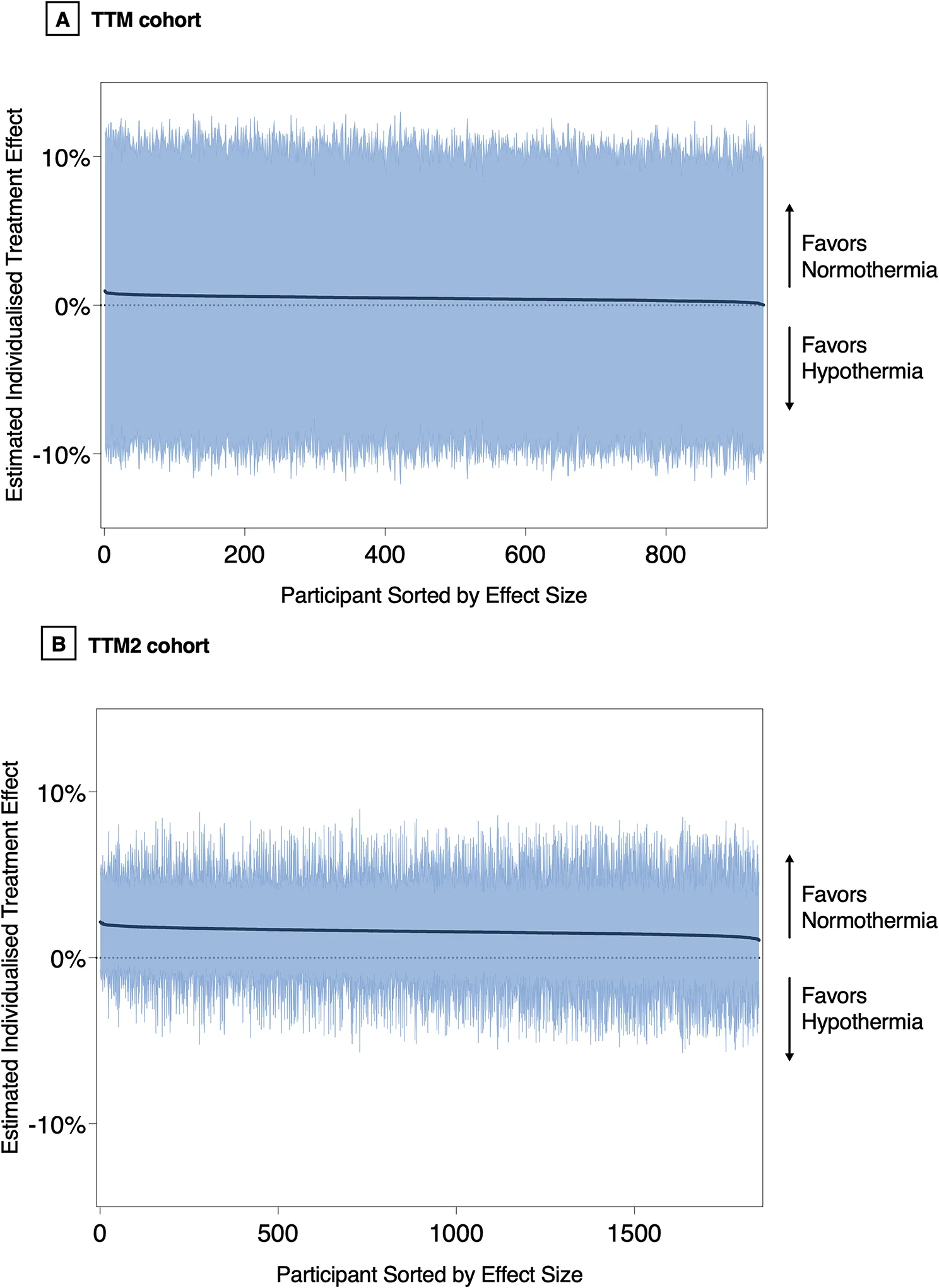
Heterogeneity of treatment effect evaluation using individualized treatment effect modeling through application of causal forests. Figures represent estimated individualized treatment effects (ITEs) for all-cause mortality at 6 months according to hypothermia for TTM (**A**) and TTM2 (**B**) trial cohorts. Solid line shows the mortality risk difference estimate (percentage points), and the shaded area represents 95% confidence intervals. Causal forests, a non-parametric ensemble learning method, was used to estimate ITEs. TTM denotes Targeted Temperature Management at 33 °C versus 36 °C after Cardiac Arrest trial; and TTM2, Hypothermia versus Normothermia after Out-of-Hospital Cardiac Arrest trial

Using causal forests, minimal variation in the estimated individual-level 6-month mortality risk differences between the treatment arms was observed in TTM (Fig. 1A) and TTM2 (Fig. 1B), suggesting no clinically meaningful HTE based on the baseline variables considered. In TTM, estimated conditional mean mortality risk differences ranged from approximately 0.02% to 0.9%, with confidence intervals that included the null for all participants (omnibus test for heterogeneity, one-sided *p* = 1.00). In TTM2, estimated conditional mean mortality risk differences ranged from approximately 1.1% to 2.2% (95% CIs including the null value), with no evidence of heterogeneity (omnibus test for heterogeneity, one-sided *p* = 1.00). Nested cross validation analyses similarly showed no evidence of heterogeneity of treatment effect (combined one-sided *p =* 0.83 for TTM, *p =* 0.23 for TTM2; Supplementary Figure S2A and S2B).

**Fig. 2:**
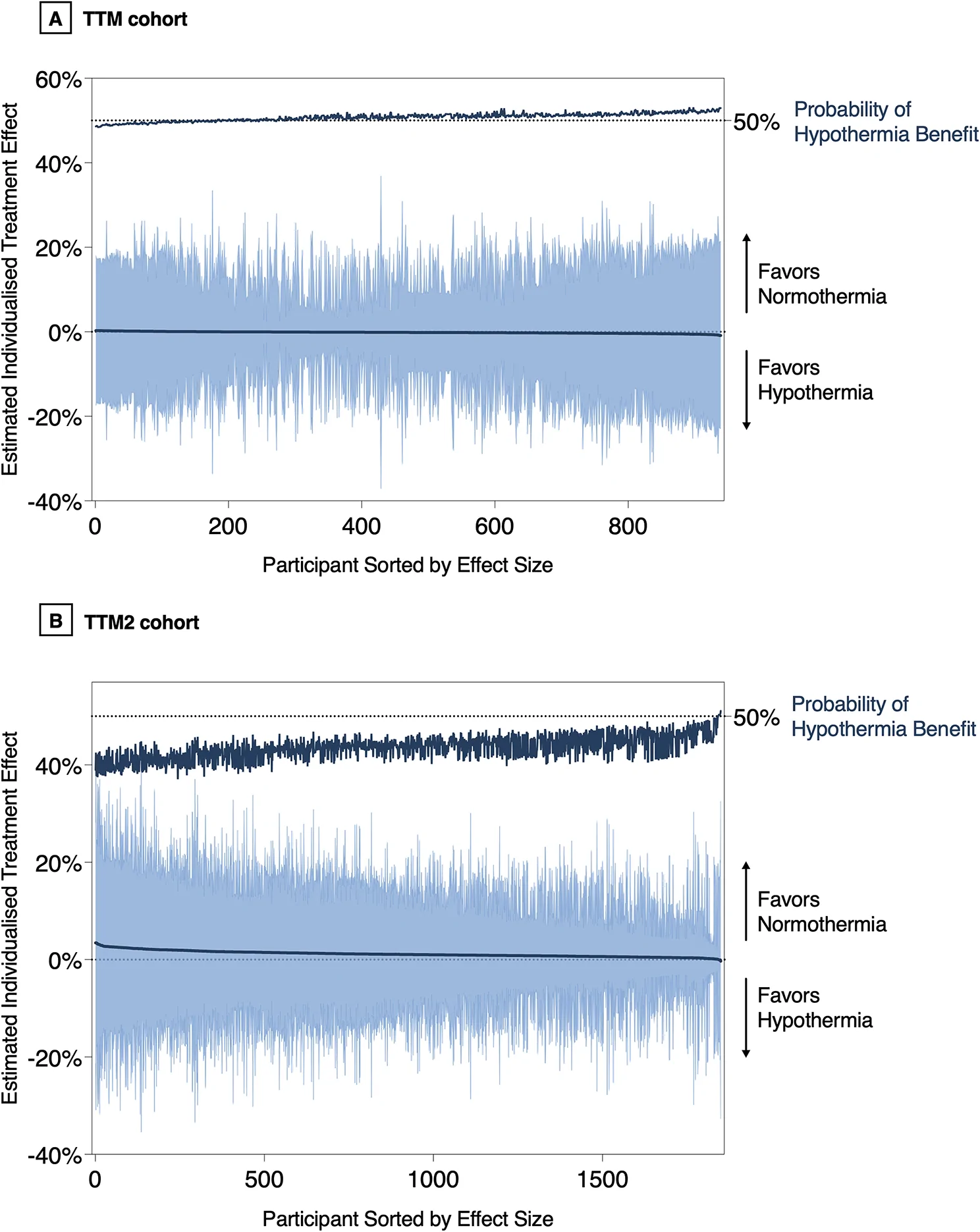
Heterogeneity of treatment effect evaluation using individualized treatment effect modeling through application of BART. Figures represent estimated individualized treatment effects (ITEs) for all-cause mortality at 6 months according to hypothermia for the TTM (**A**) and TTM2 (**B**) trial cohorts. Solid line shows the mortality risk difference estimate (percentage points), and the shaded area represents 95% credible intervals, the corresponding values are indicated on the left y-axis. Blue line above represents the estimated probability of survival benefit under hypothermia treatment, reference probability of 50% is depicted on the right y-axis. Each point reflects the posterior probability for one patient: values > 50% favor hypothermia. Bayesian Additive Regressions trees (BART), a non-parametric Bayesian ensemble method, was used to estimate ITEs and posterior probabilities. TTM denotes Targeted Temperature Management at 33 °C versus 36 °C after Cardiac Arrest trial; and TTM2, Hypothermia versus Normothermia after Out-of-Hospital Cardiac Arrest trial

Using BART, estimated conditional mortality risk differences were centered around zero for all participants (Fig. 2A and B), with wide Bayesian credible intervals that included null effect for all patients. Estimated conditional mortality risk differences ranged from approximately − 0.9% to 0.3% in TTM (Fig. 2A) and from − 0.3% to 3.5% in TTM2 (Fig. 2B). Posterior probabilities for a beneficial effect of 33 °C ranged from 49% to 53% in TTM (Fig. 2A) and from 39% to 51% in TTM2 (Fig. 2B).

### Machine learning for effect modifier detection

**Fig. 3:**
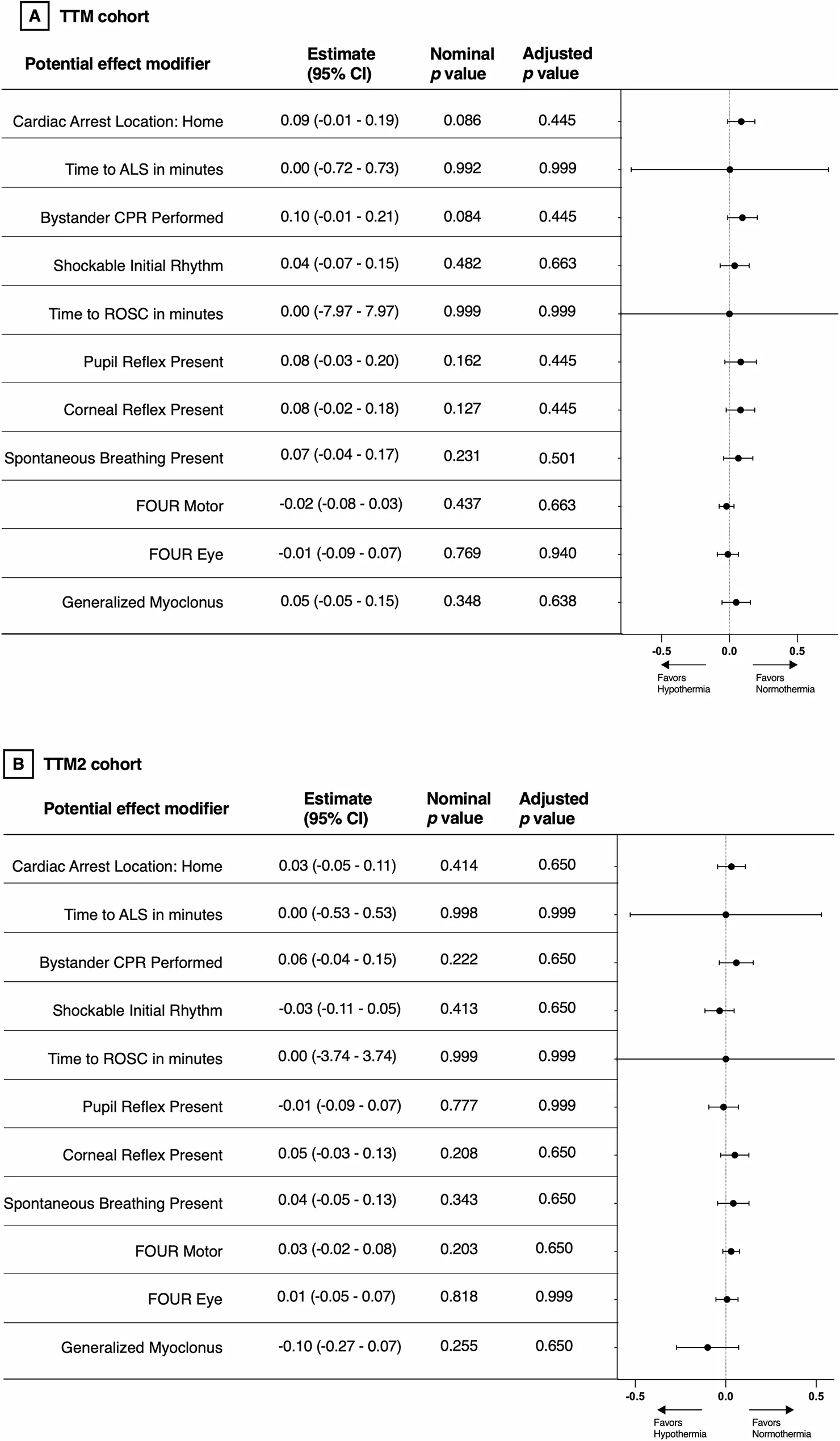
Treatment effect modifier evaluation according to potential effect modifiers through application of the TEM-VIP analysis. Figures represent the estimated change in the average treatment effect for all-cause mortality at 6 months for the TTM (**A**) and TTM2 (**B**) trial cohorts for a unit increase in the potential effect modifier. Forest plots depict the point estimates and 95% confidence intervals of the treatment effect modifier variable importance parameters (TEM-VIPs). ALS denotes advanced life support; CI, confidence interval; CPR, cardiopulmonary resuscitation; FOUR, Full Outline of UnResponsiveness Score; ROSC, return of spontaneous circulation; TEM-VIP, treatment effect modifier variable importance parameter; TTM, Targeted Temperature Management at 33 °C versus 36 °C after Cardiac Arrest trial; and TTM2, Hypothermia versus Normothermia after Out-of-Hospital Cardiac Arrest trial

We applied a machine-learning method for screening single-effect modifiers. The TEM-VIPs for the baseline clinical covariates were inferred in the TTM (Fig. 3A) and TTM2 (Fig. 3B) cohorts using a targeted maximum likelihood estimator. This analysis did not identify evidence of significant effect modification by any of the considered baseline clinical covariates. In both trials, none of the subgroup estimates were significantly different from zero at the nominal level of significance (Fig. 3).

### Secondary pooled cohort analysis

In the pooled trial cohort, which included a total of 2789 patients with OHCA, causal forest analysis suggested minimal variation in the estimated individual 6-month mortality risk differences between the treatment arms (estimated conditional mean mortality risk differences range − 1.2% to 2.1%, omnibus test for heterogeneity, one-sided *p* = 0.99; Supplementary Figure S3). The findings were consistent across the nested cross-validation analysis (combined one-sided *p =* 0.51; Supplementary Figure S2C). BART findings were centered around zero in the pooled TTM and TTM2 cohort, with individual-level mortality risk differences ranging from − 0.5% to 1.5% and posterior probabilities ranging from 42% to 53% (Supplementary Figure S4). In TEM-VIP analysis, absolute subgroup estimates were small and non-significant (Supplementary Figure S5). The largest observed effect was among patients who received bystander CPR, although this was non-significant (estimate = 0.071, 95% CI 0.00–0.14, nominal *p* = 0.051, adjusted *p* = 0.368).

### Secondary analyses

Six-month functional outcome, measured by the modified Rankin Scale, was available for 2762 (99.0%) patients (Supplementary Figure S1). Estimated individual risk differences in 6-month functional outcome using causal forests varied minimally based on the included traits (omnibus test for heterogeneity, one-sided *p* = 0.99 for TTM, *p =* 1.00 for TTM2, *p =* 0.99 for the pooled cohort, Supplementary Figure S6). Findings were consistent in nested outer cross-validation causal forest sensitivity analysis (combined one-sided *p =* 0.12 for TTM, *p =* 0.98 for TTM2, *p =* 0.74 for the pooled cohort, Supplementary Figure S7). Results were similar with BART (Supplementary Figure S8). No statistically significant potential effect modifiers for the 6-month functional outcome were identified by the TEM-VIP procedure (Supplementary Figure S9).

Among the correlates of ischemic burden and neurological variables not previously evaluated as single subgroups (cardiac arrest location, pupillary reflexes, corneal reflexes, spontaneous breathing, FOUR motor score, FOUR eye score, and generalized myoclonus), none demonstrated significant effect modification in conventional subgroup analyses for all-cause mortality or poor functional outcome at six months (Supplementary Figures S10 and S11).

Complete case sensitivity analyses (*N* = 1462) in the combined dataset of TTM and TTM2 trials demonstrated no evidence of heterogeneous treatment effects across all methods (causal forest, BART, and TEM-VIP) for all-cause mortality and poor functional outcome, consistent with the primary *missForest* imputed analyses (Supplementary Figure S12). Patients included and excluded from the complete case analysis differed in several baseline, cardiac arrest, and admission characteristics, while others were similar between groups (Supplementary Table S5). Neurological examination variables such as FOUR score and corneal reflexes had substantial missingness.

## Discussion

This secondary analysis of the TTM and TTM2 trials evaluated the hypothesis that ischemic burden correlates and initial neurological findings may identify a subset of patients who may benefit from 33 °C therapeutic hypothermia after OHCA. Effect-based machine learning was applied to probe for HTE based on these variables. Among the 2789 patients, the developed models did not identify evidence that outcomes differed from the overall trial conclusion of neutrality [3, 4] based on available ischemic burden correlates or initial neurological findings, with only limited variation in estimated treatment effects between the analyses. Results were consistent across secondary and sensitivity analyses, and with single-variable subgroup analyses. Together, these findings provide no evidence of detectable HTE based on the available clinical correlates of ischemic burden and initial neurological findings evaluated in this study.

Several factors may explain the discrepancy between the findings of the present analysis and those of prior observational studies [6–9, 11]. First, because temperature targets in observational studies were selected at the physician’s or the participating hospital’s discretion [9, 11], treatment-selection bias and residual confounding cannot be excluded, whereas randomization in the TTM trials provides a less biased estimate of the comparative effects of hypothermia and controlled normothermia. Our findings were robust across a range of advanced and conventional statistical methodologies. Second, our analysis did not include EEG data, which was a key feature defining patients associated with benefit in a previous observational study [9]. However, these ancillary studies are typically performed after the clinical decision to initiate temperature control to hypothermia. As a result, these tests cannot inform the initial decision for temperature control, and as post-randomization variables, they may themselves be influenced by the treatment, introducing potential bias.

Recent advances in critical care RCT science are shifting HTE assessment from one-variable subgroups to predictive, individualized effect modeling that integrates causal inference with machine learning, aiming to deliver more targeted therapies across heterogeneous ICU populations [14, 15, 36, 37]. Compared with conventional subgroup analyses, these approaches offer potential theoretical advantages by allowing estimation of a mean treatment effect for each patient, simultaneous evaluation of many covariates, capture of nonlinear relationships and high-order interactions between variables, empirical selection of variable cut-offs, handling of overlapping group membership, and control of multiple testing [13].

It is important to consider the findings from this study in the context of other methods for assessing possible HTE, of which there are generally three: conventional one-at-a-time subgroup analyses, risk-based HTE (where relative treatment effects vary based on risk for the predicted outcome), and effect-based HTE [38]. The findings herein are consistent with prior TTM and TTM2 analyses, which evaluated one-at-a-time subgroup effects and risk-based HTE analyses that model relative treatment effects across the range of predicted risk for a long-term poor functional outcome [3–5].

Detecting HTE remains challenging because most trials are powered for tests of average treatment effects rather than subgroup-specific differences. This difficulty is amplified in trials with overall neutral effects, where the statistical signal for detecting true heterogeneity is inherently limited even with large sample sizes, such as TTM and TTM2 trials. This motivates the application of newer analytical methods and the pooling of patient-level data to increase sample size. However, pooling requires strong assumptions that are rarely fully met [39], as illustrated by differences between TTM and TTM2 trials. Although both trials evaluated temperature control strategies after OHCA in broadly similar patient populations, differences in comparator strategies, eligibility criteria, and trial conduct mean that pooling should not be viewed as equivalent to increasing sample size for an identical treatment contrast. Accordingly, the pooled analyses are best interpreted as an exploratory assessment of whether consistent signals of treatment heterogeneity can be identified across related, but not identical, trial settings. While this reduces interpretability, it underscores the need for harmonized trial designs and patient-level meta-analyses to enable robust individualized effect modeling in future research. In addition to these inherent challenges, advanced effect-based modeling methods face risks of overfitting, substantial sample size requirements, model convergence issues in smaller cohorts [40], and constraints related to data availability [41]. Through growing adoption, the methods and their limitations can be increasingly better understood.

Interpretation of treatment heterogeneity is further influenced by how accurately candidate effect modifiers reflect the underlying biological process of interest. The neurological examination variables evaluated as potential effect modifiers may not directly reflect the severity of hypoxic-ischemic brain injury. Although neurological assessments were obtained on hospital admission in both trials, early post-arrest examination findings may still be influenced by sedation, neuromuscular blockade, and hemodynamic instability [42–44]. Furthermore, neurological assessments were not performed identically across trials, requiring harmonization of Glasgow Coma Scale-based assessments from TTM with FOUR score categories recorded in TTM2. The variables examined as clinical correlates of ischemic burden should be viewed as indirect measures of ischemia-reperfusion injury, as patients with similar times to ALS or ROSC may experience different no-flow and low-flow durations. This study aimed to test the hypothesis that patients with clinical correlates suggesting greater ischemic burden may derive differential benefit from lower temperature targets. Consequently, measurement error, imperfect characterization of the underlying injury process, and the exclusion of other potentially relevant clinical characteristics may have attenuated true treatment effect modification and reduced the ability to detect clinically meaningful HTE.

This work has several potential limitations. First, this analysis was *post hoc*, and findings should be regarded as exploratory and hypothesis-generating. Additionally, subgroups that exhibit HTE may not have been adequately captured by the selected covariates or represented within the trials’ population. Second, missing data were handled using a non-parametric single-imputation approach (*missForest*), which cannot fully account for imputation uncertainty. Although patients included and excluded from the complete case sensitivity analysis differed in some measured characteristics, no consistent pattern of greater or lesser illness severity was observed, and mortality, functional outcome, and treatment allocation were similar between groups. Nevertheless, informative missingness cannot be excluded and may have influenced HTE estimates, even if complete case sensitivity analyses yielded results consistent with the primary analysis. Third, the pooled analysis should be interpreted with caution because TTM and TTM2 trials differed with respect to comparator strategies, eligibility criteria, and trial conduct, limiting the extent to which pooling can be considered equivalent to increasing sample size for a single treatment comparison. Fourth, candidate variables reflecting the clinical question were selected based on available RCT data and may not fully capture the complexity of brain injury severity. As noted, however, the ancillary testing findings are typically unavailable at admission, and clinically meaningful prognostic findings generally manifest beyond the initial hours in which randomization occurs. Future research may consider evaluating and interrogating additional neurological markers, such as EEG, biomarkers of brain injury, and neuroimaging in future RCTs. Although the causal forest and BART models can accommodate complex non-linear treatment-effect relationships, TEM-VIP may be less sensitive to non-monotonic patterns of effect modification. Finally, by virtue of the TTM and TTM2 inclusion criteria, this exploratory analysis excludes patients with presumed non-cardiac causes of arrest, in-hospital cardiac arrest, or those treated with strategies other than hypothermic temperature control. The normothermia arms in TTM and TTM2 trials involved active temperature control and strict fever prevention, rather than permissive normothermia without device-based intervention, meaning the trials cannot determine whether either strategy is superior to an approach without active temperature management.

## Conclusions

In this *post hoc* secondary analysis of the TTM and TTM2 trials, we found no evidence of detectable heterogeneity of treatment effect between targeted temperature of 33 °C and controlled normothermia based on the available baseline clinical characteristics, including correlates of ischemic burden and initial neurological examination findings. Across all analytical approaches, the estimated variation in treatment effects was small.

Tables.

## Supplementary Information

Below is the link to the electronic supplementary material.

Supplementary Material 1

## Data Availability

The datasets used and analysed during the current study are available from the corresponding author on reasonable request.

## Abbreviations

ALS: Advanced life support
BART: Bayesian additive regression trees
CPR: Cardiopulmonary resuscitation
EEG: Electroencephalogram
FOUR: Full outline of unresponsiveness score
HTE: Heterogeneous treatment effect
ITE: Individualized treatment effect
OHCA: Out-of-hospital cardiac arrest
RCT: Randomized clinical trial
ROSC: Return of spontaneous circulation
TEM-VIP: Treatment effect modifier variable importance parameter
TTM: Target temperature management at 33 °C versus 36 °C after cardiac arrest trial
TTM2: Hypothermia versus normothermia after out-of-hospital cardiac arrest trial
