# Supplementary material for: Differentiated effect of hypothermia versus normothermia after cardiac arrest by neurological status and clinical correlates of ischemic burden
Source: Crit Care. 2026 Nov 2;30:561. doi: 10.1186/s13054-026-06414-x (PMC13729339; doi:10.1186/s13054-026-06414-x)
Supplement: Supplementary file 1 [file 13054_2026_6414_MOESM1_ESM.docx]

Supplementary Material

**Differentiated effect of hypothermia versus normothermia after cardiac arrest by neurological status and clinical correlates of ischemic burden**

Gabriele Lileikyte MD,^1,2^ Lucas L. Marinho MD PhD,^3^ Ying Lu MS,^3^ Philippe Boileau PhD,^4^ Marion Moseby-Knappe MD PhD,^5,6^ Alexandra Spicer MS,^7^ Josef Dankiewicz MD PhD,^8,9^ Gisela Lilja PhD,^10,11^ Matt P. Wise Dphil,^12^ Claudia Schrag MD,^13^ Anders Aneman,^14^ Matthias Haenggi MD,^15^ Anders M. Grejs MD PhD,^16^ Janneke Horn,^17^ Pascal Stammet MD PhD,^18,19^ Janus C. Jakobsen PhD,^20,21^ Jesper Kjaergaard,^22,23^ Christian Rylander MD PhD,^24^ Michael O. Harhay PhD MPH,^25^ Matthew M. Churpek MD PhD MPH,^7^ Niklas Nielsen MD PhD,^26,27^* Patrick R. Lawler MD MPH^3,28^*

* Equal contribution

**Corresponding author:** Gabriele Lileikyte, gabriele.lileikyte@med.lu.se, Department of Clinical Sciences Helsingborg, Helsingborg hospital, Svartbrödragränden 3, 251 87

Table of Contents

[Supplementary Methods	3](#_Toc242184728)

[Variable Selection and Harmonization	3](#_Toc242184729)

[Missing Data Imputation	4](#_Toc242184730)

[Effect-based Individualized Treatment Effect Estimation using Causal Forests	5](#_Toc242184731)

[Effect-based Individualized Treatment Effect Estimation using Bayesian Additive Regression Trees	7](#_Toc242184732)

[Ensemble Machine Learning for Effect Modifier Detection	8](#_Toc242184733)

[Conventional Subgroup Analysis	9](#_Toc242184734)

[Supplementary Tables and Figures	10](#_Toc242184735)

[Table S1.	10](#_Toc242184736)

[Table S2.	11](#_Toc242184737)

[Table S3.	12](#_Toc242184738)

[Table S4.	13](#_Toc242184739)

[Table S5.	15](#_Toc242184740)

[Figure S1.	17](#_Toc242184741)

[Figure S2.	18](#_Toc242184742)

[Figure S3.	20](#_Toc242184743)

[Figure S4.	21](#_Toc242184744)

[Figure S5.	22](#_Toc242184745)

[Figure S6.	23](#_Toc242184746)

[Figure S7.	25](#_Toc242184747)

[Figure S8.	27](#_Toc242184748)

[Figure S9.	29](#_Toc242184749)

[Figure S10.	32](#_Toc242184750)

[Figure S11.	34](#_Toc242184751)

[Figure S12.	36](#_Toc242184752)

[References	40](#_Toc242184753)

# Supplemental Methods

## Variable Selection and Harmonization

To enable pooled analyses, variables were harmonized prior to combining the Target Temperature Management at 33°C versus 36°C after Out-of-Hospital Cardiac Arrest (TTM) and Hypothermia versus Normothermia after Out-of-Hospital Cardiac Arrest (TTM2) trials’ datasets (1, 2). Location of cardiac arrest was recorded into a binary variable, with public place classified as “other” in both datasets. First monitored rhythm was categorized as binary; shockable rhythms included non-perfusing ventricular tachycardia, return of spontaneous circulation (ROSC) after bystander defibrillation, unknown rhythm with shock administered, and ventricular fibrillation. Non-shockable rhythms included pulseless electrical activity, asystole, and unknown rhythm with no shock administered. Admission pupillary and corneal reflexes were recorded as binary variables in TTM and as categorical variables in TTM2 according to Full Outline of UnResponsiveness (FOUR) score (3); unilateral responses in TTM2 were recorded as “present” to match the binary format. Spontaneous breathing was defined in TTM as any sign of breathing, including agonal breathing or gasping. In TTM2, breathing was assessed using the FOUR score and therefore modified to binary for the analysis: FOUR respiration patterns score 0 was recorded as “absent” and scores 1-4 as “present”. Motor (M) and eye (E) responses were recorded using the Glasgow Coma Scale (GCS) score in TTM and the FOUR score in TTM2. GCS values were mapped to FOUR categories: GCS-M 1 to FOUR-M 0, GCS-M 2 to FOUR-M 1, GCS-M 3–4 to FOUR-M 2, GCS-M 5 to FOUR-M 3, and GCS-M 6 to FOUR-M 4. GCS-E 1 was mapped to FOUR-E 0, GCS-E 2 to FOUR-E 1, GCS-E 3 to FOUR-E 2, and GCS-E 4 to FOUR-E 4. Sedation-related entries in TTM (29% for GCS-M and 30% for GCS-E) were categorized as non-assessed, as this category was not present in TTM2. For conventional subgroup analyses, FOUR eye and motor scores were dichotomized. FOUR eye score was divided into score 0 (no eye opening) and scores 1-4 (eye opening to pain, loud voice, or spontaneously with or without tracking). FOUR motor score was dichotomized to scores 1-2 (pathological extension or flexion) and scores 0, 3-4 (no motor response, localizing to pain, or spontaneous movement) as no motor response might have been affected by sedation in the TTM2 trial. Generalized myoclonus was defined in TTM as convulsions in the face and extremities lasting at least 30 minutes within the first 24 hours. In TTM2, myoclonus was assessed at hospital admission and recorded as “no”, “yes, generalized”, or “yes, not generalized”. To match the TTM definition, “yes, not generalized” was recorded as “no”. A variable indicating the data source (TTM or TTM2 trial) was included in the combined dataset to account for trial origin in the Causal Forest and Bayesian Additive Regression Trees analyses.

## Missing Data Imputation

Missing values were imputed using a non-parametric method based on Random Forests (*missForest* algorithm) that accommodates both continuous and categorical variables without distributional assumptions (4). To optimize performance of the *missForest* algorithm, a custom cross-validation procedure, where a fraction (10%) of the observed values in the variables with missing data was randomly masked and treated as missing, was used to evaluate combinations of hyperparameters (number of trees, iterations, and variables per split). The configuration with the lowest classification error and out-of-bag error was selected for final imputation. Imputation was performed separately for each dataset to preserve dataset-specific distributions and missingness mechanisms. Across the TTM dataset, missingness ranged from 0.1% to 30.6% across the 11 variables, while in TTM2 it ranged from 6.9% to 43.6% across 6 variables (**Supplementary Table S2**). Overall, 7.7% and 16.9% of all values were imputed in TTM and TTM2 datasets, respectively.

Multiple imputation was not used because integrating it with the primary estimators (Bayesian Additive Regression Trees Analysis (BART) and causal forests including nested cross-validation workflow) would require fold-specific re-imputation and nontrivial pooling of nonparametric functionals, increasing the risk of data leakage and model incompatibility. In this setting, multiple imputation would entail re-imputation within each training fold across inner resampling loops and hyperparameter configurations, with limited methodological guidance for valid pooling. To account for this, complete case sensitivity analyses were performed by restricting the dataset to individuals with available data for all variables included in the heterogeneity of treatment effect analyses. Baseline characteristics and outcomes were compared between patients included and excluded from the complete case sensitivity analysis. Continuous variables were presented as medians with interquartile ranges and compared using the Wilcoxon rank-sum test. Categorical variables were presented as counts with percentages and compared using Pearson’s chi-squared test or Fisher’s exact test, as appropriate. The causal forest, BART, and TEM-VIP analyses were re-estimated on this subset from the combined TTM and TTM2 trial dataset.

## Effect-based Individualized Treatment Effect Estimation using Causal Forests

Causal forests are a non-parametric ensemble learning method that extend the random forest (5) framework to infer treatment effect heterogeneity (6, 7). To detect HTE, causal forests build an ensemble of decision trees that recursively partition the covariate space into regions where treatment effects differ, using sample-splitting and orthogonalization to obtain asymptomatically unbiased estimates of conditional average treatment effects (CATEs). Individual-level CATEs are estimated as smooth functions of baseline covariates via forest-based weighting of scores; aggregation across trees stabilizes predictions and captures complex effect modification. Throughout the manuscript, model-derived patient-level CATE estimates were referred to as individualized treatment effect (ITE) estimates. This terminology is intended to distinguish such estimates from the true individual treatment effect, which is fundamentally unobservable (8). The causal forest model was implemented using the generalized random forests framework (*grf* R package, version 2.4.0) (7). ITE estimates were generated directly from the fitted forest as a function of the baseline clinical covariates. The treatment propensity score was fixed at 0.5 for all participants, reflecting the arm randomization probabilities. Model hyperparameters were tuned using the *grf* tuning procedure for all tunable model hyperparameters (“sample.fraction”, “mtry”, “min.node.size”, “honesty.fraction”, “honesty.prune.leaves”, “alpha”, “imbalance.penalty”; tune.parameters = "all") (6). ITE estimates were reported on the risk difference scale (33°C minus normothermia, negative values indicate benefit of 33°C) with 95% confidence intervals (CI) derived from the model-based variance estimates. To formally evaluate whether the forest identified meaningful treatment-effect variation, we applied an omnibus test of the causal forest’s calibration to evaluate the HTE hypothesis (9).

To assess the robustness and generalizability of the estimated individual treatment effects, we performed nested cross-validation of the causal forest models. A 10-fold outer cross-validation procedure was applied to obtain out-of-sample CATE estimates. For each outer fold, a causal forest was trained on the remaining folds, and participant-level CATE predictions and variance estimates were generated exclusively from the held-out fold. Rank-based measures of heterogeneity and treatment effect prioritization were subsequently evaluated on the held-out data using rank-weighted average treatment effect (RATE) metrics with Qini weighting. Fold-specific inference was aggregated using the Aggregated Cauchy Association Test (ACAT) to evaluate the HTE hypothesis (9).

## Effect-based Individualized Treatment Effect Estimation using Bayesian Additive Regression Trees

To robustly assess for HTE, we employed a second analytic framework to estimate ITE. This approach used BART, a non-parametric Bayesian regression method that models outcomes as a sum of many small regression trees under a regularization prior, enabling flexible, data-driven function estimation without strong parametric assumptions (10). BART was selected for its theoretical advantages including quantifying uncertainty through credible intervals, reducing overfitting via regularization priors, and competitive predictive accuracy without parametric assumptions. BART was implemented using a custom parallelized framework based on the *lbart* function from the *BART* R package (version 2.9.9) (11). Treatment assignment was included as a covariate alongside the predefined baseline clinical covariates, and mean counterfactual outcomes were predicted by setting the treatment indicator to 33°C and normothermia for each participant. ITE estimates along with their 95% credible intervals were then produced by computing the difference of each patients predicted outcome under treatment at 33°C and normothermia.

BART prior hyperparameters were selected from a 10-fold cross-validation, comparing predictive performance of every combination of power priors equal to 1, 2, or 3, base priors equal to 0.25, 0.5, or 0.95, and number of trees equal to 50, 200, or 400, to minimize out-of-sample prediction error. The priors that corresponded to the lowest prediction error from the cross validation were applied to final models (**Supplementary Table S3**). Posterior Markov Chain Monte Carlo (MCMC) samples which consisted of four chains, number of burn-in iterations and total length of iterations, were used to draw inference (**Supplementary Table S3**). Convergence of MCMC chains was assessed using Geweke diagnostics (12). Estimated ITEs were calculated as the difference in posterior means (33°C minus normothermia, with negative values indicating benefit of 33°C) with 95% credible intervals, and the posterior probability of hypothermia benefit (ITE <0) was computed for each individual. HTE was evaluated by examining the spread of posterior means and their credible intervals and determining whether the posterior probability variation exceeded what would be expected under a constant treatment effect.

## Ensemble Machine Learning for Effect Modifier Detection

As a further analysis to probe for relevant effect modifiers across the range of baseline neurological and ischemia-duration correlated variables, we performed a treatment effect modifier variable importance parameter (TEM-VIP) analysis (13, 14). This causal machine learning framework enables the non-parametric quantification of individual baseline covariates’ capacity for effect modification on the absolute and relative scales, in turn permitting the formal testing and ranking of candidate effect modifiers. The absolute scale TEM-VIPs of the baseline clinical covariates were estimated, along with their 95% confidence intervals, using a targeted maximum likelihood estimator (15, 16) implemented in the *unihtee* R package (version 0.03). Importantly, multiplicity-adjusted tests to evaluate whether the absolute TEM-VIPs were significantly different from zero were performed; non-zero absolute TEM-VIPs imply effect modification and therefore a heterogeneous treatment effect. A Super Learner ensemble (17) was employed to estimate conditional mean outcomes, incorporating penalized regression (lasso, ridge, elastic net) (18, 19), XGboost, random forests, and multivariate adaptive regression splines (20). Interaction terms between clinical covariates and treatment assignment were manually defined in the penalized regression and multivariate adaptive regression spline models to enhance detection of treatment effect modification.

## Conventional Subgroup Analysis

A prior individual patient data meta-analysis of the TTM and TTM2 trial populations evaluated treatment interactions between time to ROSC, time to advanced life services, witnessed arrest, bystander CPR, and initial rhythm (21). For subgroup analysis, we used the remaining, not-previously evaluated, variables (cardiac arrest location, pupillary reflexes, corneal reflexes, spontaneous breathing, FOUR motor score, FOUR eye score, and generalized myoclonus). Subgroup analyses were conducted using Poisson regression with a log link function to estimate risk ratios (RR) and 95% confidence intervals (CI) (22). Robust standard errors were applied to account for potential overdispersion. To assess potential effect modification, interaction terms between treatment group and each subgroup variable were included in the Poisson regression models. Robust standard errors were used to calculate interaction p-values. Subgroup analyses were exploratory and not adjusted for multiple comparisons.

# Supplemental Tables and Figures

Table S1. Inclusion and exclusion criteria for TTM and TTM2 trials.

| **Trial** | **Inclusion criteria** | **Exclusion criteria** |
| --- | --- | --- |
| TTM | - Age ≥18 years. - Out of hospital cardiac arrest of presumed cardiac cause. - Sustained return of spontaneous circulation (ROSC) for more than 20 consecutive minutes after resuscitation - Unconsciousness (GCS <8) (patients not able to obey verbal commands) after sustained ROSC. | - Obvious or suspected pregnancy - Known bleeding diathesis (medically induced coagulopathy (e.g. warfarin, clopidogrel does not exclude the patient). - Suspected or confirmed acute intracranial bleeding - Suspected or confirmed acute stroke - Unwitnessed cardiac arrest with initial rhythm asystole - Known limitations in therapy and Do Not Resuscitate-order - Known disease making 180 days survival unlikely - Known pre-arrest Cerebral Performance Category 3 or 4 - >4 hours (240 minutes) from ROSC to - screening - Systolic blood pressure <80 mmHg despite fluid loading/vasopressor and/or inotropic medication/intra-aortic balloon pump. If the systolic blood pressure was recovering during the inclusion window (220 minutes), the patient could be included. - Temperature on admission <30°C. |
| TTM2 | - Out-of-hospital cardiac arrest of a presumed cardiac or unknown cause - Sustained Return of spontaneous circulation (ROSC) - defined as 20 minutes with signs of circulation without the need for chest compressions. - Unconsciousness defined as not being able to obey verbal commands (FOUR-score motor response of <4) and no verbal response to pain after sustained ROSC. - Eligible for intensive care without restrictions or limitations - Inclusion within 180 minutes of ROSC. | - Unwitnessed cardiac arrest with an initial rhythm of asystole - Temperature on admission <30°C. - On Extracorporeal Membrane Oxygenation prior to ROSC - Obvious or suspected pregnancy - Intracranial bleeding - Severe chronic obstructive pulmonary disorder (COPD) with long-term home oxygen therapy. |

TTM denotes Targeted Temperature Management at 33°C versus 36°C after Cardiac Arrest trial; TTM2, Hypothermia versus Normothermia after Out-of-Hospital Cardiac Arrest trial; ROSC, Return of spontaneous circulation; GCS, Glasgow coma scale; FOUR-score, Full outline of unresponsiveness score; COPD, Chronic obstructive pulmonary disease.

Table S2. Proportion of missing values for imputed variables in the TTM and TTM2 datasets.

| **Variable** | **Missing (%) in TTM trial** | **Missing (%) in TTM2 trial** |
| --- | --- | --- |
| Cardiac arrest location | 0.1 | 0 |
| Time to ALS in minutes | 1.8 | 0 |
| Bystander CPR | 0.2 | 0 |
| Initial rhythm | 0.1 | 0 |
| Time to ROSC in minutes | 0.1 | 0 |
| Pupil reflex | 2.6 | 17.4 |
| Corneal reflex | 15.2 | 43.6 |
| Spontaneous breathing | 4.0 | 6.9 |
| Motor response, FOUR | 29.6 | 8.9 |
| Eye Response, FOUR | 30.6 | 9.7 |
| Generalized myoclonus | 0.3 | 14.7 |

TTM denotes Targeted Temperature Management at 33°C versus 36°C after Cardiac Arrest trial; TTM2, Hypothermia versus Normothermia after Out-of-Hospital Cardiac Arrest trial; ALS, advanced life support; CPR, cardiopulmonary resuscitation; ROSC, return of spontaneous circulation; FOUR, Full Outline of Unresponsiveness Score.

Table S3. Cross-validation results for Bayesian Additive Regression Trees (BART) analysis according to cohort.

| **Cohort** | **Prior Hyperparameters** | **Markov Chain Monte Carlo (MCMC)** |
| --- | --- | --- |
| *All-cause mortality at 6 months* | | |
| TTM | Power prior = 1  Base prior = 0.25  Number of trees = 400 | Burn-in iterations n = 5000  Total iterations n = 15000 |
| TTM2 | Power prior = 1  Base prior = 0.5  Number of trees = 400 | Burn-in iterations n = 10000  Total iterations n = 15000 |
| Combined cohort of TTM and TTM2 | Power prior = 1  Base prior = 0.25  Number of trees = 400 | Burn-in iterations n = 5000  Total iterations n = 15000 |
| *Poor functional outcome at 6 months (modified Rankin scale score of 4 to 6)* | | |
| TTM | Power prior = 3  Base prior = 0.25  Number of trees = 400 | Burn-in iterations n = 5000  Total iterations n = 15000 |
| TTM2 | Power prior = 2  Base prior = 0.25  Number of trees = 400 | Burn-in iterations n = 10000  Total iterations n = 15000 |
| Combined cohort of TTM and TTM2 | Power prior = 2  Base prior = 0.25  Number of trees = 400 | Burn-in iterations n = 5000  Total iterations n = 15000 |

TTM denotes Targeted Temperature Management at 33°C versus 36°C after Cardiac Arrest trial; TTM2, Hypothermia versus Normothermia after Out-of-Hospital Cardiac Arrest trial.

Table S4. Patient characteristics according to treatment group. *

|  | **Hypothermia 33 °C**  **(N = 1398)** | **Normothermia (N = 1391)** |
| --- | --- | --- |
| Age in years | 66.0 (57.0-73.0) | 65.0 (55.0-73.0) |
| Male sex | 1131 (80.9%) | 1097 (78.9%) |
| **Medical history** (known/previous) | | |
| Heart failure | 122/1357 (9.0%) | 121/1340 (9.0%) |
| Acute myocardial infarction | 246/1358 (18.1%) | 238/1340 (17.8%) |
| Coronary-artery bypass grafting | 120/1358 (8.8%) | 118/1338 (8.8%) |
| Hypertension | 537/1358 (39.5%) | 479/1338 (35.8%) |
| **Cardiac arrest** | | |
| Cardiac arrest at home | 732/1398 (52.4%) | 745/1390 (53.6%) |
| Minutes to ALS † | 10.0 (6.0-15.0) | 10.0 (6.0-14.0) |
| Bystander CPR performed | 1099/1398 (78.6%) | 1061/1389 (76.3%) |
| Initial rhythm shockable | 1041/1398 (74.5%) | 1071/1390 (75.7%) |
| Minutes to ROSC | 25.0 (17.0-40.0) | 25.0 (17.0-40.0) ‡ |
| **Clinical characteristics on admission** | | |
| Pupillary reflexes present | 880/1215 (72.4%) | 896/1228 (73.0%) |
| Corneal reflexes present | 437/915 (47.8%) | 456/924 (49.4%) |
| Spontaneous breathing present | 523/1319 (39.7%) | 528/1304 (40.4%) |
| Motor response, FOUR § |  |  |
| No response to pain (0) | 975 (69.7%) | 968 (69.6%) |
| Extension response to pain (1) | 47 (3.4%) | 466 (3.3%) |
| Flexion response to pain (2) | 105 (7.5%) | 115 (8.3%) |
| Localizing to pain (3) | 42 (3.0%) | 42 (3.0%) |
| Thumbs-up, fist, or peace sign (4) | 5 (0.4%) | 1 (0.1%) |
| Eye response, FOUR ** |  |  |
| Eyelids remain closed with pain (0) | 1096 (78.4%) | 1088 (78.2%) |
| Eyelids closed but open to pain (1) | 30 (2.1%) | 28 (2.0%) |
| Eyelids closed but open to loud voice (2) | 3 (0.2%) | 6 (0.4%) |
| Eyelids open but not tracking (3) | 24 (1.7%) | 29 (2.1%) |
| Eyelids open and tracking (4) | 13 (0.9%) | 5 (0.4%) |
| Generalized myoclonus present | 39/1259 (3.1%) | 39/1255 (3.1%) |
| Initial temperature (°C) †† | 35.4 (34.7-36.0) | 35.5 (34.8-36.1) |
| pH ‡‡ | 7.22 (7.10-7.30) | 7.22 (7.11-7.29) |
| Lactate mmol/l §§ | 5.2 (2.7-8.5) | 5.2 (2.6-8.7) |
| Circulatory shock | 330/1397 (23.6%) | 341/1390 (24.5%) |
| ST-segment elevation myocardial infarction | 565/1379 (41.0%) | 561/1377 (40.7%) |

* Results reported as numbers (percentages) or median (interquartile range), as appropriate. TTM denotes Targeted Temperature Management at 33°C versus 36°C after Cardiac Arrest trial; TTM2, Hypothermia versus Normothermia after Out-of-Hospital Cardiac Arrest trial; ALS, advanced life support; CPR, cardiopulmonary resuscitation; ROSC, return of spontaneous circulation; FOUR, Full Outline of Unresponsiveness Score.

† Missing values in 8 patients in the hypothermia group, in 9 patients in the normothermia group.

‡ Missing value in one patient.

§ Missing values in 224 patients in the hypothermia group and 219 patients in the normothermia group.

** Missing values in 232 patients in the hypothermia group and 235 patients in the normothermia group.

†† Missing values in 177 patients in the hypothermia group and 159 patients in the normothermia group.

‡‡ Missing values in 39 patients in the hypothermia group and 39 patients in the normothermia group.

§§ Missing values in 66 patients in the hypothermia group and 76 patients in the normothermia group.

Table S5. Characteristics of patients included in and excluded from the complete case sensitivity analysis.*

|  | **Included in complete case sensitivity analysis**  **(N = 1462)** | **Excluded from complete case sensitivity analysis (N = 1327)** | ***P* value*** |
| --- | --- | --- | --- |
| Age in years | 65.0 (56.0-73.8) | 66.0 (56.0-73.0) | 0.4 |
| Male sex | 1176 (80.4%) | 1052 (79.3%) | 0.4 |
| **Medical history** (known/previous) | | |  |
| Heart failure | 138/1425 (9.7%) | 105/1272 (8.3%) | 0.2 |
| Acute myocardial infarction | 276/1425 (19.4%) | 208/1273 (16.3%) | **0.041** |
| Coronary-artery bypass grafting | 131/1425 (9.2%) | 107/1271 (8.4%) | 0.5 |
| Hypertension | 530/1426 (37.2%) | 486/1270 (38.3%) | 0.6 |
| **Cardiac arrest** | | |  |
| Cardiac arrest at home | 775 (53.0%) | 702/1326 (52.9%)  (1 value missing) | >0.9 |
| Minutes to ALS | 10.0 (6.0-14.0) | 10.0 (6.0-15.0)  (17 values missing) | 0.7 |
| Bystander CPR performed | 1124 (76.9%) | 1036/1325 (78.2%)  (2 values missing) | 0.4 |
| Initial rhythm shockable | 1089 (74.5%) | 1023/1326 (77.1%)  (1 value missing) | 0.1 |
| Minutes to ROSC | 25.0 (16.0-38.8) | 26.0 (17.0-40.0)  (1 value missing) | **0.017** |
| **Clinical characteristics on admission** | | |  |
| Pupillary reflexes present | 1004 (68.7%) | 772/981 (78.7%)  (346 values missing) | **<0.001** |
| Corneal reflexes present | 680 (47.0%) | 213/377 (56.5%)  (950 values missing) | **<0.001** |
| Spontaneous breathing present | 633 (43.0%) | 418/1161 (36.0%)  (166 values missing) | **<0.001** |
| Motor response, FOUR |  | (443 values missing) | 0.069 |
| No response to pain (0) | 1198 (81.9%) | 745 (84.2%) |  |
| Extension response to pain (1) | 54 (3.7%) | 39 (4.4%) |  |
| Flexion response to pain (2) | 153 (10.5%) | 67 (7.6%) |  |
| Localizing to pain (3) | 55 (3.8%) | 29 (3.3%) |  |
| Thumbs-up, fist, or peace sign (4) | 2 (0.1%) | 4 (0.5%) |  |
| Eye response, FOUR |  | (467 values missing) | 0.065 |
| Eyelids remain closed with pain (0) | 1382 (94.5%) | 802 (93.3%) |  |
| Eyelids closed but open to pain (1) | 36 (2.5%) | 22 (2.6%) |  |
| Eyelids closed but open to loud voice (2) | 6 (0.4%) | 3 (0.3%) |  |
| Eyelids open but not tracking (3) | 24 (1.6%) | 29 (3.4%) |  |
| Eyelids open and tracking (4) | 14 (1.0%) | 4 (0.5%) |  |
| Generalized myoclonus present | 43 (2.9%) | 35/1052 (3.3%)  (275 values missing) | 0.6 |
| Initial temperature (°C) | 35.5 (34.8-36.1)  (117 values missing) | 35.4 (34.7-36.1)  (219 values missing) | 0.1 |
| pH | 7.22 (7.10-7.29)  (44 values missing) | 7.22 (7.11-7.30)  (34 values missing) | 0.3 |
| Lactate mmol/l | 5.6 (2.9-8.9)  (75 values missing) | 4.7 (2.4-8.2)  (67 values missing) | **<0.001** |
| Circulatory shock | 346 (23.7%) | 325/1327 (24.5%)  (2 values missing) | 0.6 |
| ST-segment elevation myocardial infarction | 585/1448 (40.4%)  (14 values missing) | 541/1308 (41.4%)  (19 values missing) | 0.6 |
| Hypothermia as randomization group | 734 (50.2%) | 664 (50.0%) | >0.9 |
| Dead at six months | 723 (49.5%) | 634 (47.8%) | 0.4 |
| Poor functional outcome (mRS 4-6) at six months | 783 (54%) | 689/1300 (53.0%)  (27 values missing) | 0.8 |

* Patients were classified as included if complete data were available for all variables used for the heterogeneity of treatment analyses and excluded otherwise. Results reported as numbers (percentages) or median (interquartile range), as appropriate. *P* values were calculated using the Wilcoxon rank-sum test for continuous variables and Pearson’s chi-squared test or Fisher’s exact test, as appropriate.

Figure S1. CONSORT flow chart for Targeted Temperature Management at 33°C versus 36°C after Cardiac Arrest (TTM) trial and Hypothermia versus Normothermia after Out-of-Hospital Cardiac Arrest (TTM2) trial.

Analysed

TTM: n = 469 (n = 4 not possible to contact)

TTM2: n = 918 (n = 7 assessment not possible)

Analysed

TTM: n = 464 (n = 2 did not want to attend

follow-up visit)

TTM2: n = 911 (n = 14 assessment not

possible)

Analysed

TTM: n = 466 (n = 3 no recorded cardiac

arrest, n = 2 non-cardiac cause of arrest)

TTM2: n = 925 (n = 6 not possible to contact

and no records)

Analysed

TTM: n = 473 (n = 1 limitations in care, n = 1 awake)

TTM2: n = 925 (n = 5 not possible to contact and no records)

Analysed

TTM: n = 471 (n = 3 consent withdrawn)

TTM2: n = 931 (n = 1 double randomisation, n = 19 consent not obtained or withdrawn)

Randomized:

TTM: n = 950

TTM2: n = 1900

Assessed for eligibility:

TTM: n = 1431

TTM2: n = 4355

***Analysis***

***Allocation***

Analysed

TTM: n = 475 (n = 1 consent withdrawn)

TTM2: n = 930 (n = 1 double randomisation, n = 18 consent not obtained or withdrawn)

Allocated to Normothermia / 36 °C

TTM: n = 474

TTM2: n = 951

Allocated to Hypothermia

TTM: n = 476

TTM2: n = 949

***Enrollment***

***Functional Outcome Assessed***

***at 6-months***

***Mortality Assessed***

***at 6-months***

TTM Excluded (n = 481)

- Not meeting inclusion criteria (n = 189)
- Other reasons, exclusion criteria (n = 292)

TTM2 Excluded (n = 2455)

- Not meeting inclusion criteria (n = 1909)
- Other reasons, exclusion criteria (n = 546)

Figure S2. Heterogeneity of Treatment Effect Evaluation using Individualized Treatment Effect (ITE) Modelling through application of Causal forests with nested outer cross-validation. Figures represent estimated ITEs for all-cause mortality at 6 months according to hypothermia for the TTM (A)* and TTM2 (B)** trial cohorts as well as the combined TTM and TTM2 (C) trial cohort. Solid line shows the mortality risk difference estimate (percentage points), and the shaded area represents 95% confidence intervals. ACAT combined one-sided *p =* 0.83 for TTM (A), *p =* 0.23 for TTM2 (B), *p =* 0.51 for combined TTM and TTM2 (C) cohort.

TTM denotes Targeted Temperature Management at 33°C versus 36°C after Cardiac Arrest.

* Certain folds in the figure 2A display uniform treatment-effect estimates. This reflects the causal forest converging to a constant prediction in folds where minimal or no heterogeneity is present.

**Figure S2 cont.**

TTM denotes Targeted Temperature Management at 33°C versus 36°C after Cardiac Arrest; and TTM2, Hypothermia versus Normothermia after Out-of-Hospital Cardiac Arrest.

** A certain fold in the figure 2B displays uniform treatment-effect estimates. This reflects the causal forest converging to a constant prediction in folds where minimal or no heterogeneity is present.

Figure S3. Heterogeneity of Treatment Effect Evaluation using Individualized Treatment Effect (ITE) Modelling through application of Causal forests. Figure represents estimated ITEs for all-cause mortality at 6 months according to hypothermia for the combined TTM and TTM2 trial cohort. Solid line shows the mortality risk difference estimate (percentage points), and the shaded area represents 95% confidence intervals. Causal forests, a non-parametric ensemble learning method, was used to estimate ITEs.

TTM denotes Targeted Temperature Management at 33°C versus 36°C after Cardiac Arrest; and TTM2, Hypothermia versus Normothermia after Out-of-Hospital Cardiac Arrest.

Figure S4. Heterogeneity of Treatment Effect Evaluation using Individualized Treatment Effect (ITE) Modelling through application of Bayesian Additive Regression Trees (BART). The figure shows estimated ITEs for all-cause mortality at 6 months according to hypothermia for the combined TTM and TTM2 trial cohort. Solid line shows the mortality risk difference estimate (percentage points), and the shaded area represents 95% credible intervals, the corresponding values are indicated on the left y-axis. Blue line above represents the estimated probability of survival benefit under hypothermia treatment, reference probability of 50% is depicted on the right y-axis. Each point reflects the posterior probability for one patient: values >50% favor hypothermia. BART, a non-parametric Bayesian ensemble method, was used to estimate ITEs and posterior probabilities.

TTM denotes Targeted Temperature Management at 33°C versus 36°C after Cardiac Arrest; and TTM2, Hypothermia versus Normothermia after Out-of-Hospital Cardiac Arrest.

Figure S5.Treatment effect modifier evaluation according to potential effect modifiers through application of the treatment effect modifier variable importance parameter (TEM-VIP) analysis. Figure represents the estimated change in the average treatment effect for all-cause mortality at 6 months for the combined TTM and TTM2 trial cohort for a unit increase in the potential effect modifier. Forest plots depict the point estimates and 95% confidence intervals of the TEM-VIPs.

ALS denotes advanced life support; CI, confidence interval; CPR, cardiopulmonary resuscitation; FOUR, Full Outline of UnResponsiveness Score; ROSC, return of spontaneous circulation; TTM, Targeted Temperature Management at 33°C versus 36°C after Cardiac Arrest; and TTM2, Hypothermia versus Normothermia after Out-of-Hospital Cardiac Arrest.

Figure S6. Heterogeneity of Treatment Effect Evaluation using Individualized Treatment Effect (ITE) Modelling through application of Causal forests. Figures show estimated ITEs for poor functional outcome (modified Rankin Scale score 4 to 6) at 6 months according to hypothermia for the TTM (A) and TTM2 (B) trial cohorts as well as the combined TTM and TTM2 (C) trial cohort. Solid line shows the risk difference estimate for poor functional outcome (percentage points), and the shaded area represents 95% confidence intervals. Causal forests, a non-parametric ensemble learning method, was used to estimate ITEs. Results for Omnibus test for heterogeneity; TTM trial - one-sided *p* = 0.99, TTM2 trial - one-sided *p* = 1.00, combined TTM and TTM2 cohort - one-sided *p* = 0.99.

TTM denotes Targeted Temperature Management at 33°C versus 36°C after Cardiac Arrest.

**Figure S6 cont.**

TTM denotes Targeted Temperature Management at 33°C versus 36°C after Cardiac Arrest; and TTM2, Hypothermia versus Normothermia after Out-of-Hospital Cardiac Arrest.

Figure S7. Heterogeneity of Treatment Effect Evaluation using Individualized Treatment Effect (ITE) Modelling through application of Causal forests with nested outer cross-validation. Figures show estimated ITEs for poor functional outcome (modified Rankin Scale score 4 to 6) at 6 months according to hypothermia for the TTM (A) and TTM2 (B)* trial cohorts as well as the combined TTM and TTM2 (C) trial cohort. Solid line shows the risk difference estimate for poor functional outcome (percentage points), and the shaded area represents 95% confidence intervals. Results for ACAT combined one-sided *p =* 0.12 for TTM (A), *p =* 0.98 for TTM2 (B), *p =* 0.74 for combined TTM and TTM2 (C) cohort.

TTM denotes Targeted Temperature Management at 33°C versus 36°C after Cardiac Arrest.

**Figure S7 cont.**

TTM denotes Targeted Temperature Management at 33°C versus 36°C after Cardiac Arrest; and TTM2, Hypothermia versus Normothermia after Out-of-Hospital Cardiac Arrest.

* Certain folds in the figure 7B display uniform treatment-effect estimates. This reflects the causal forest converging to a constant prediction in folds where minimal or no heterogeneity is present.

Figure S8. Heterogeneity of Treatment Effect Evaluation using Individualized Treatment Effect (ITE) Modelling through application of Bayesian Additive Regressions trees (BART). Figures show estimated ITEs for poor functional outcome (modified Rankin Scale score 4 to 6) at 6 months according to hypothermia for the TTM (A) and TTM2 (B) trial cohorts as well as the combined TTM and TTM2 (C) trial cohort. Solid line shows the risk difference estimate for poor functional outcome (percentage points), and the shaded area represents 95% credible intervals, the corresponding values are indicated on the left y-axis. Blue line above represents the estimated probability of good outcome under hypothermia treatment, reference probability of 50% is depicted on the right y-axis. Each point reflects the posterior probability for one patient: values >50% favor hypothermia. BART, a non-parametric Bayesian ensemble method, was used to estimate ITEs and posterior probabilities.

TTM denotes Targeted Temperature Management at 33°C versus 36°C after Cardiac Arrest.

**Figure S8 cont.**

TTM denotes Targeted Temperature Management at 33°C versus 36°C after Cardiac Arrest; and TTM2, Hypothermia versus Normothermia after Out-of-Hospital Cardiac Arrest.

Figure S9. Treatment effect modifier evaluation according to potential effect modifiers through application of the treatment effect modifier variable importance parameter (TEM-VIP) analysis. Figures represent the estimated change in the average treatment effect for poor functional outcome (modified Rankin Scale score 4 to 6) at 6 months for the TTM (A) and TTM2 (B) trial cohorts as well as the combined TTM and TTM2 (C) trial cohort for a unit increase in the potential effect modifier. Forest plots depict the point estimates and 95% confidence intervals of the TEM-VIPs.

ALS denotes advanced life support; CI, confidence interval; CPR, cardiopulmonary resuscitation; FOUR, Full Outline of UnResponsiveness Score; ROSC, return of spontaneous circulation; and TTM, Targeted Temperature Management at 33°C versus 36°C after Cardiac Arrest.

**Figure S9 cont.**

ALS denotes advanced life support; CI, confidence interval; CPR, cardiopulmonary resuscitation; FOUR, Full Outline of UnResponsiveness Score; ROSC, return of spontaneous circulation; and TTM2, Hypothermia versus Normothermia after Out-of-Hospital Cardiac Arrest.

**Figure S9 cont.**

ALS denotes advanced life support; CI, confidence interval; CPR, cardiopulmonary resuscitation; FOUR, Full Outline of UnResponsiveness Score; ROSC, return of spontaneous circulation; TTM, Targeted Temperature Management at 33°C versus 36°C after Cardiac Arrest; and TTM2, Hypothermia versus Normothermia after Out-of-Hospital Cardiac Arrest.

Figure S10. Heterogeneity of Treatment Effect Evaluation by Conventional Subgroup Analysis. Figure represents risk ratios for all-cause mortality at 6 months stratified by subgroup for the TTM (A) and TTM2 (B) trial cohorts as well as the combined TTM and TTM2 (C) trial cohort. Subgroup analyses were conducted using Poisson regression with a log link function to estimate risk ratios (RR) and 95% confidence intervals (CI). Interaction terms between treatment group and each subgroup variable were included in the model to assess potential effect modification. Numbers of patients at risk are depicted for the imputed cohorts.

CI denotes confidence interval; FOUR, Full Outline of UnResponsiveness Score; and TTM, Targeted Temperature Management at 33°C versus 36°C after Cardiac Arrest.

**Figure S10 cont.**

CI denotes confidence interval; FOUR, Full Outline of UnResponsiveness Score; TTM, Targeted Temperature Management at 33°C versus 36°C after Cardiac Arrest; and TTM2, Hypothermia versus Normothermia after Out-of-Hospital Cardiac Arrest.

Figure S11. Heterogeneity of Treatment Effect Evaluation by Conventional Subgroup Analysis. Figures represent risk ratios for poor functional outcome (modified Rankin Scale score 4 to 6) at 6 months stratified by subgroup for the TTM (A) and TTM2 (B) trial cohorts as well as the combined TTM and TTM2 (C) trial cohort. Subgroup analyses were conducted using Poisson regression with a log link function to estimate risk ratios (RR) and 95% confidence intervals (CI). Interaction terms between treatment group and each subgroup variable were included in the model to assess potential effect modification. Numbers of patients at risk are depicted for the imputed cohorts.

CI denotes confidence interval; FOUR, Full Outline of UnResponsiveness Score; and TTM, Targeted Temperature Management at 33°C versus 36°C after Cardiac Arrest.

**Figure S11 cont.**

CI denotes confidence interval; FOUR, Full Outline of UnResponsiveness Score; TTM, Targeted Temperature Management at 33°C versus 36°C after Cardiac Arrest; and TTM2, Hypothermia versus Normothermia after Out-of-Hospital Cardiac Arrest.

Figure S12. Complete case sensitivity analysis in the combined TTM and TTM2 trial cohort. The figures display results for causal forest analysis for all-cause mortality (A) and poor functional outcome (B); nested cross validation (CV) causal forest analysis for all-cause mortality (C) and poor functional outcome (D); Bayesian Additive Regression Trees (BART) analysis for all-cause mortality (E) and poor functional outcome (F); and treatment effect modifier variable importance parameter (TEM-VIP) analysis for all-cause mortality (G) and poor functional outcome (H).

TTM denotes Targeted Temperature Management at 33°C versus 36°C after Cardiac Arrest; and TTM2, Hypothermia versus Normothermia after Out-of-Hospital Cardiac Arrest.

CV denotes cross validation; TTM, Targeted Temperature Management at 33°C versus 36°C after Cardiac Arrest; and TTM2, Hypothermia versus Normothermia after Out-of-Hospital Cardiac Arrest.

TTM denotes Targeted Temperature Management at 33°C versus 36°C after Cardiac Arrest; and TTM2, Hypothermia versus Normothermia after Out-of-Hospital Cardiac Arrest.

ALS denotes advanced life support; CI, confidence interval; CPR, cardiopulmonary resuscitation; FOUR, Full Outline of UnResponsiveness Score; ROSC, return of spontaneous circulation; and TTM2, Hypothermia versus Normothermia after Out-of-Hospital Cardiac Arrest.

# References

1.	Nielsen N, Wetterslev J, Cronberg T, Erlinge D, Gasche Y, Hassager C, et al. Targeted temperature management at 33°C versus 36°C after cardiac arrest. New England Journal of Medicine. 2013;369(23):2197–206.

2.	Dankiewicz J, Cronberg T, Lilja G, Jakobsen JC, Levin H, Ullén S, et al. Hypothermia versus Normothermia after Out-of-Hospital Cardiac Arrest. New England Journal of Medicine. 2021;384(24):2283–94.

3.	Wijdicks EFM, Bamlet WR, Maramattom BV, Manno EM, McClelland RL. Validation of a new coma scale: The FOUR score. Annals of Neurology. 2005;58(4):585–93.

4.	Stekhoven DJ, Bühlmann P. MissForest—non-parametric missing value imputation for mixed-type data. Bioinformatics. 2012;28(1):112–8.

5.	Breiman L. Random Forests. Machine Learning. 2001;45(1):5–32.

6.	Wager S, and Athey S. Estimation and Inference of Heterogeneous Treatment Effects using Random Forests. Journal of the American Statistical Association. 2018;113(523):1228–42.

7.	Athey S, Tibshirani J, Wager S. Generalized random forests. The Annals of Statistics. 2019;47(2):1148–78.

8.	Munroe ES, Spicer A, Castellvi-Font A, Zalucky A, Dianti J, Graham Linck E, et al. Evidence-based personalised medicine in critical care: a framework for quantifying and applying individualised treatment effects in patients who are critically ill. The Lancet Respiratory Medicine. 2025;13(6):556–68.

9.	Chernozhukov V, Demirer M, Duflo E, Fernández-Val I. Generic machine learning inference on heterogeneous treatment effects in randomized experiments, with an application to immunization in India. arXiv preprint. 2018;arXiv:1712.04802.

10.	Chipman HA, George EI, McCulloch RE. BART: Bayesian additive regression trees. The Annals of Applied Statistics. 2010;4(1):266–98, 33.

11.	Sparapani R, Spanbauer C, McCulloch R. Nonparametric Machine Learning and Efficient Computation with Bayesian Additive Regression Trees: The BART R Package. Journal of Statistical Software. 2021;97(1):1 – 66.

12.	Geweke J. Evaluating the Accuracy of Sampling-Based Approaches to the Calculation of Posterior Moments. In: Bernardo J, Berger J, Dawid A, Smith A, editors. Bayesian Statistics. 4. Oxford: Oxford University Press; 1992. p. 166–93.

13.	Boileau P, Qi NT, van der Laan MJ, Dudoit S, Leng N. A flexible approach for predictive biomarker discovery. Biostatistics. 2022;24(4):1085–105.

14.	Boileau P, Leng N, Hejazi NS, van der Laan M, Dudoit S. A nonparametric framework for treatment effect modifier discovery in high dimensions. Journal of the Royal Statistical Society Series B: Statistical Methodology. 2024;87(1):157–85.

15.	van der Laan MJ, Rubin D. Targeted Maximum Likelihood Learning. The International Journal of Biostatistics.2(1).

16.	van der Laan MJ, Rose S. Targeted learning : causal inference for observational and experimental data. New York ;: Springer; 2011.

17.	van der Laan MJ, Polley EC, Hubbard AE. Super Learner. Statistical Applications in Genetics and Molecular Biology.6(1).

18.	Zhu J, Hastie T. Classification of gene microarrays by penalized logistic regression. Biostatistics. 2004;5(3):427–43.

19.	Zou H, Hastie T. Regularization and Variable Selection Via the Elastic Net. Journal of the Royal Statistical Society Series B: Statistical Methodology. 2005;67(2):301–20.

20.	Jerome HF. Multivariate Adaptive Regression Splines. The Annals of Statistics. 1991;19(1):1–67.

21.	Holgersson J, Meyer Martin Abild S, Dankiewicz J, Lilja G, Ullén S, Hassager C, et al. Hypothermic versus Normothermic Temperature Control after Cardiac Arrest. NEJM Evidence. 2022;1(11):EVIDoa2200137.

22.	Zou G. A Modified Poisson Regression Approach to Prospective Studies with Binary Data. American Journal of Epidemiology. 2004;159(7):702–6.
